# Haemophagocytic lymphohistiocytosis (HLH) following allogeneic haematopoietic stem cell transplantation (HSCT)—time to reappraise with modern diagnostic and treatment strategies?

**DOI:** 10.1038/s41409-019-0637-7

**Published:** 2019-08-27

**Authors:** Robert David Sandler, Stuart Carter, Harpreet Kaur, Sebastian Francis, Rachel Scarlett Tattersall, John Andrew Snowden

**Affiliations:** 1 Department of Rheumatology, Sheffield Teaching Hospitals NHS Foundation Trust, Royal Hallamshire Hospital, Sheffield, S10 2JF UK; 2 Department of Haematology, Sheffield Teaching Hospitals NHS Foundation Trust, Royal Hallamshire Hospital, Sheffield, S10 2JF UK

**Keywords:** Haematological cancer, Inflammatory diseases, Graft-versus-host disease

## Introduction

Haemophagocytic lymphohistiocytosis (HLH) is a hyperinflammatory condition, characterised by inappropriate survival of histiocytes and cytotoxic T-lymphocytes (CTL), which, if undiagnosed and untreated, leads to cytokine storm and haemophagocytosis [1]. HLH affects children, adolescents and adults, leading to multi-organ failure and high mortality. HLH can be may be familial (fHLH) or secondary/acquired (sHLH).

FHLH usually presents in infancy due to inherited defects in cytolytic proteins, is classified using the HLH-2004 criteria, and treated with immunosuppression (HLH-2004 protocol) to “bridge” patients to definitive treatment with haematopoietic stem cell transplantation (HSCT) [2].

SHLH is triggered by malignancy, infection or autoimmunity. There are no definitive diagnostic criteria for sHLH in adults. Diagnosis is often extrapolated from fHLH criteria (HLH-2004) or the relatively novel “*H* score” [2, 3]. Treatment is similarly extrapolated from the HLH-2004 immunosuppressive regimen [2, 4]. Recent advances in the understanding of rheumatological sHLH led to specific classification criteria for systemic juvenile idiopathic arthritis (sJIA)-associated sHLH, with treatment protocols advocating interleukin-1 (IL-1) blockade [5]. Serum ferritin is recognised as a cheap and accessible biomarker with levels of ≥10,000 μg/ml considered highly suggestive of HLH in febrile, unwell patients [6]. This has prompted further rheumatological consideration and proposal of diagnostic and therapeutic approaches to sHLH, beyond the confines of sJIA [7]. There are no published guidelines for modern diagnostic and therapeutic approaches to sHLH in the adult post-HSCT population, where mortality remains high.

Here we appraise the literature and summarise six cases of sHLH following adult HSCT over a 5-year period (2014–18), suggesting sHLH is an under-recognised post-HSCT complication.

## Pathogenesis of HLH

HLH is characterised by inappropriate survival of histiocytes and failure of normal cytolytic functions of natural killer (NK) cells and CTL. Inability to clear antigens from infection, malignant cells or autoimmune/autoinflammatory processes leads to inappropriate immune stimulation. This predisposes the hyperinflammatory state, or cytokine storm, in which innate immune system dysfunction is a key, and IL-1 is central to pathogenesis [8–13].

In fHLH and related immunodeficiency syndromes, inherited cytolytic defects, particularly concerning perforin, impair NK cell and CTL activity [10]. Susceptibility to HLH arises from uncontrolled cellular proliferation and survival, when the immune response is triggered, for example by infection [14]. The genetic basis of fHLH is increasingly well recognised and treatment outlined in the HLH-2004 protocol [2]. Emapalumab, a novel interferon-γ antagonist, has recently been approved for fHLH and efficacy in sHLH is being investigated [15–17]. FHLH is diagnosed in mostly infancy and early childhood but there are reports of diagnosis in adulthood [18].

SHLH comprises a heterogeneous group of hyperinflammatory syndromes occurring when the “hyperinflammatory threshold” is breached by interplay of genetic predisposition and triggers such as infection, malignancy and inflammation [7, 10]. Although patients with sHLH can be genetically predisposed, in contrast to fHLH, non-genetic triggers play a greater role in reaching the threshold [19]. Therefore, long-term remission, or even “cure”, may be achieved with targeted treatment strategies without using the HLH-2004 protocol or rescue HSCT.

## sHLH and haematological malignancy

sHLH is most commonly associated with haematological malignancies, such as T cell and NK-cell leukaemia, diffuse large B-cell lymphoma and Hodgkin lymphoma [7]. Here, sHLH is likely driven by the malignant pro-inflammatory state, but contemporaneous Epstein–Barr virus (EBV), can be a contributory factor [20, 21]. SHLH has been identified in up to 10% of patients undergoing chemotherapy for acute myeloid leukaemia (AML) [22].

## sHLH and infection

In adults, the leading cause of sHLH worldwide is viral infection, with EBV the predominant trigger in the USA and Asia [23]. Other herpes viruses, including cytomegalovirus (CMV), herpes simplex (HSV) and varicella zoster (VZV) are common triggers with human immunodeficiency virus, influenza, dengue and ebola also recognised [24–27].

## sHLH and autoimmune disease (MAS)

sHLH is termed macrophage activation syndrome (MAS) when associated with rheumatological disease. MAS is well recognised in sJIA, where infections, particularly EBV or VZV, are acknowledged triggers [28, 29]. Defects in genes coding for perforin, similar to those seen in fHLH, are reported in sJIA and associated with development of MAS [19]. In adults, it is most prevalent (up to 15%) in adult onset Still’s disease, which is considered within the same spectrum as sJIA [30]. A retrospective study identified MAS in one third of systemic lupus erythematosus (SLE) patients admitted to hospital with fever, with associated tenfold rise in mortality [31]. Whilst MAS has been identified in other rheumatological conditions, it is thought that preceding infection or immunosuppression, rather than the pathophysiology of the autoimmune condition, are likely triggering factors [32–34].

## sHLH post-HSCT

A 10-year retrospective Japanese survey identified 42 cases of sHLH post-HSCT in children, associated with 59% mortality in the event of non-resolution versus 15% in cases with resolution [35]. Patients with acute lymphoblastic leukaemia (*n* = 12), AML (*n* = 6), aplastic anaemia (*n* = 4), immunodeficiency (*n* = 4), juvenile monomyelocytic leukaemia (*n* = 3), chronic leukaemia (*n* = 2) or alternative malignancies (*n* = 11) underwent autologous, allogeneic or umbilical cord HSCT, though distribution within these groups was not reported. A 5-year study in Japanese adults undergoing HSCT identified an sHLH incidence of 4.3%, with 85.5% mortality [36]. Twenty-five percent underwent autologous and 75% allogeneic HSCT. Reports of death due to MAS in patients with refractory JIA undergoing autologous HSCT, led to changes in immunosuppressive and infectious prophylactic regimens, leading to decreased mortality [37].

A prospective, single-centre Tunisian study of paediatric and adult cases of sHLH post-HSCT identified an overall incidence of 4%, increasing to 8.8% when reviewing allogeneic alone [38]. Of seven cases of sHLH, three were attributed to CMV, one to EBV and three had no known aetiology. The investigators propose that the higher incidence following allogenic HSCT may indicate that sHLH is a form of GvHD, in that activation of host macrophages, in response to donor stem cells, give features of GvHD, similar those of sHLH [35, 38]. Case reports support the assertion that sHLH may be an allogeneic response to donor cells, in the early stages post-HSCT with no identified viral trigger [39, 40]. Colita et al. report a patient with lymphoma, with haemophagocytosis seen on bone marrow biopsy on day +22 post-HSCT. Unable to identify a donor for a rescue allogeneic HSCT, they commenced intravenous immunoglobulin (IVIG), resulting in cure. Despite developing multiple myeloma, as a second malignancy, there was no evidence of active sHLH at 8 months [41].

## Diagnosis of HLH

Diagnosing HLH requires a high index of clinical suspicion in at-risk patients, as features overlap those of severe sepsis or malignancy, with fever and multi-organ failure. Persistent fever in patients without an identified cause, or worsening fever in patients who have been treated for infection, should prompt consideration of sHLH [42].

Laboratory values may be within normal limits and can be less helpful in isolation than a review of trends. Equally important are poor prognostic indicators of HLH, including neurological dysfunction, acute kidney injury and acute respiratory distress [7]. Serum ferritin is a useful, readily available biomarker of sHLH presence and response to treatment [38, 43–45]. In a single-centre retrospective paediatric review, serum ferritin ≥10 000 μg/L was 96% specific and 90% sensitive for HLH [6]. Serum ferritin is closely related to disease activity, and both maximum levels during sHLH, and a fall of <50% after treatment are associated with higher mortality [46–48]. Furthermore, serial ferritin measurement is useful to monitor response, as a fall to baseline is observed with successful treatment, and rebounds in recurrence [49].

In the HSCT context, ferritin levels are not strongly associated with presence of GvHD, so may prove a useful biomarker allowing differentiation from sHLH [50, 51]. Furthermore, the widespread availability, low cost and contribution to assessment of treatment response, mean the usefulness in the assessment of patients with suspected HLH cannot be overstated [52]. However ferritin remains a non-specific biomarker and several studies in the literature have reported high ferritin levels in association with transfusion, liver disease/hepatocellular injury, infection, haematological malignancy and renal failure more often than HLH [53].

Soluble IL-2 receptor (sIL-2r) has shown promise in diagnosing HLH and has greater area under the receiving operator characteristic curve than serum ferritin [54]. It is an early marker of T-cell activation and high levels are associated with worse prognosis. Furthermore, it can be used as a dynamic disease activity marker with levels expected to fall in remission, and has been demonstrated to help differentiate HLH from mimics of HLH [53–55]. That sIL-2r reflects T-cell activation whereas ferritin reflects macrophage activation presents an opportunity for tailored therapeutics if measured concurrently [55, 56]. sIL-2r and NK cell activity were added to existing parameters in the updated HLH-2004 criteria (Table 1), though have not been included in more recent criteria, such as the *H* score (Table 2) [2, 3]

**Table 1:** Classification criteria for fHLH [2]

HLH-2004 classification criteria for fHLH [2]
(1) Fever
(2) Splenomegaly
(3) Cytopenia affecting >2 lineages: haemoglobin <9 g/L, platelets < 100 × 10^9^/L, neutrophils < 1.0 × 10^9^/L
(4) Hypertriglyceridaemia and/or hypofibrinogenemia: triglycerides > 265 mg/dL, fibrinogen < 150 mg/dL
(5) Haemophagocytosis in bone marrow, spleen or lymph node
(6) Low or absent NK cell activity
(7) Ferritin > 500 μg/L
(8) Soluble cluster of differentiation (CD) 25 i.e. soluble IL-2 receptor > 2400 U/mL

**Table 2:** Classification criteria for MAS in sJIA [4]

Classification criteria for MAS in known or suspected sJIA [4]
Ferritin > 684ng/mL and two of the following
(1) Platelets < 181 × 109/L
(2) Aspartate aminotransferase (AST) > 48 U/L
(3) Triglycerides > 156 mg/dL

.

Whilst not widely available, measurement of soluble cytokines may emerge as an alternative diagnostic parameter in post-HSCT HLH, as with other HLH groups [45, 54, 57].

Classification criteria exist for HLH in some contexts. fHLH criteria (the HLH-2004 criteria) are shown in Table 1. Separate criteria exist for sHLH/MAS in patients with sJIA (Table 3) [5]. Amendments to the HLH-2004 criteria for fHLH, taking into account observations of delayed engraftment in patients who develop sHLH following umbilical cord HSCT, have been proposed (Table 4) [58]. A diagnostic calculator, the “*H* score”, takes into account clinical and laboratory features to calculate a percentage probability of sHLH in adults (Table 2) [3]. Forty-three percent of patients used to validate the “*H* Score” had underlying haematological malignancy, mostly lymphoma, but it is unclear if any had already undergone HSCT. There have been two recently proposed diagnostic (and treatment) algorithms proposed for sHLH, which are broadly similar [7, 59]. They rely on a high index of clinical suspicion and utilise readily available bedside and serological tests. Given the lack of validated diagnostic criteria for sHLH in adult patients in general, and post-HSCT patients in particular, we take a pragmatic approach to the recognition of HLH reflecting these proposed diagnostic algorithms, utilising the “*H* score” whilst recognising its limitations. Where post-HSCT patients are unwell, febrile, with a serum ferritin of ≥10000 μg/L and no proven infection (other than recognised triggers of HLH such as EBV and other herpes viral reactivations/infections seen in post-HSCT patients) they likely have hyperinflammation and should be considered for aggressive immunosuppression, as per published recommendations [6, 7, 28].

**Table 3:** Diagnostic criteria for post-HSCT HLH [51]

Diagnostic criteria for sHLH post-HSCT (adapted from HLH-2004)	
Major criteria	Minor criteria
Engraftment delay, primary or secondary failure	Fever
Histopathological evidence of haemophagocytosis	Hepatosplenomegaly
	Elevated ferritin
	Elevated lactate dehydrogenase

**Table 4:** Classification criteria for HLH (*H* Score) [3]

*H* score classification criteria for HLH [3]	
Parameter	No. of points (criteria for scoring)
Known underlying immunosuppression	0 (no) or 18 (yes)
Temperature (celcius)	0 (<38.4), 33 (38.4–39.4) or 49 (>39.4)
Organomegaly	O (no), 23 (hepatomegaly or splenomegaly) or 38 (hepatomegaly and splenomegaly)
No. of cytopenia	0 (1 lineage), 24 (2 lineages) or 34 (3 lineages)
Ferritin (µg/L)	0 (<2 000), 35 (2000–6000) or 50 (>6 000)
Triglycerides (mmol/L)	0 (<1.5), 44 (1.5–4.0) or 64 (>4.0)
Fibrinogen (g/L)	0 (≥2.5) or 30 (<2.5)
AST (IU/L)	0 (<30) or 19 (>30)
Haemophagocytosis on bone marrow aspirate	0 (no) or 35 (yes)

Inputting the above variables into the freely-available on-line calculator, found at http://saintantoine.aphp.fr/score/, produces a numerical value, the “*H* score” and subsequent percentage probability of sHLH. An “*H* score” of 169 is proposed by this group as the best cutoff to diagnose sHLH in adults, with a sensitivity of 93% and specificity of 89%

Histological identification of haemophagocytosis is recognised as a late feature of HLH and does not correlate as well as fever or serum ferritin with clinical diagnosis of sHLH [26, 60, 61]. Therefore demonstration of haemophagocytosis is not considered essential for diagnosis. In our experience haemophagocytosis may only present in the latter of serial bone marrow samples.

## Treatment of sHLH

Effective treatment of sHLH requires aggressive immunosuppression to control the hyperinflammatory state, in combination with targeted treatment against triggering factors. Prompt recognition and treatment is important and reduces mortality [62]. In clinical practice, this approach is extrapolated to sHLH following allogeneic HSCT, but its effectiveness is not currently evidence based. Broadly, the principles of treatment of HLH in the pre- and post-HSCT setting are similar, although post-HSCT HLH is considerably more complex as diagnosis and management needs to be considered amidst other post-transplant complications.

The first prospective treatment protocol for fHLH was developed in 1994 (HLH-94) before being updated as HLH-2004. HLH-94 supported a therapeutic strategy of combination chemotherapy with etoposide and immunotherapy with ciclosporin (CSA) for children with fHLH, as a bridge to definitive HSCT [2, 63]. This included 8 weeks of etoposide and dexamethasone with intrathecal methotrexate for persistent central nervous system dysfunction at 2 weeks, followed by CSA maintenance. Due to early deaths from active HLH using the HLH-94 protocol, HLH-2004 recommended more aggressive immunosuppression with first-line CSA [2].

Corticosteroids remain the cornerstone of induction treatment in sHLH, although over half of patients may be steroid-resistant [33]. Dramatic responses are reported with the addition of CSA in doses of 2–7 mg/kg/day [64]. Anakinra, an IL-1 antagonist, is effective in refractory sHLH and safe in patients with sepsis, which often features in the differential, deterring clinicians from immunosuppression [65]. Anakinra is now at the forefront of treatment in sJIA-triggered sHLH [66]. IVIG are also effective in steroid-resistant and EBV-triggered sHLH [67]. Rituximab improves overall clinical outcomes and is an important part of EBV clearance in the context of EBV-triggered sHLH or EBV-driven malignancies [21, 68]. Case reports of refractory sHLH note complete responses with rabbit anti-thymocyte globulin (ATG) or DEP regimen (doxorubicin, etoposide and methylprednisolone) and partial responses with alemtuzumab [69].

**Fig. 1:**
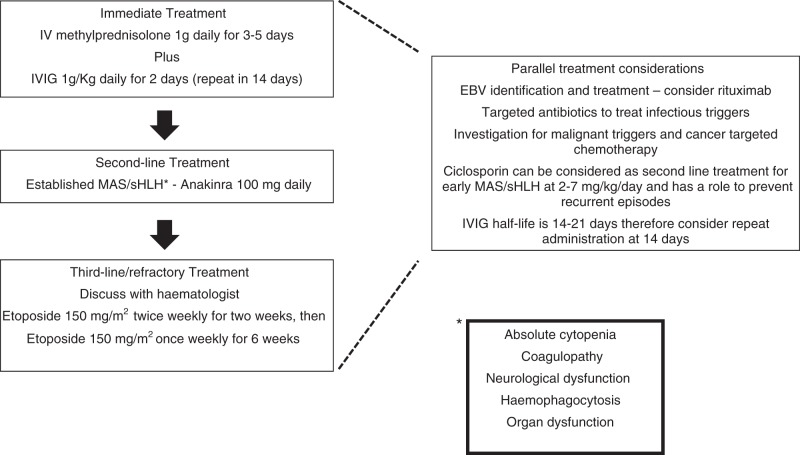
Treatment protocol for sHLH adapted with permission from Carter et al. [7]

We recently published a treatment protocol for sHLH accepting the heterogeneity of this syndrome and irrespective of preceding HSCT (Fig. 1) [7]. First-line treatment is with intravenous methylprednisolone (IVMP) 1 g/day for 3–5 days plus IVIG 1 g/kg for 2 days, which can be repeated at day 14 [70]. If there is evidence of established sHLH or clinical deterioration, IL-1 blockade with anakinra is added, 1–2 mg/kg daily increasing up to 8 mg/kg/day until sufficient clinical response. CSA is considered for early or steroid-resistant disease. Etoposide should be considered in refractory cases. There should be parallel consideration of identifying and eradicating triggers, such as EBV, bacterial infection and underlying malignancy, particularly lymphoma. A consensus treatment approach has also recently been proposed with an algorithm directing treatment according to the clinical contact and trigger of HLH. This algorithm extrapolates the HLH 2004 approach but also recognises the importance of IL-1 blockade [4]. There are no validated guidelines for treating sHLH post-HSCT and there are concerns about using the HLH-2004 protocol, especially with the inclusion of etoposide [71].

## Case series

To add to the literature, we summarise six cases of sHLH identified following allogeneic HSCT in adult patients transplanted in our unit over a 5-year period, during which we adopted early screening practice (including monitoring of ferritin) and a “rheumatological” management approach. This was a retrospective service evaluation using de-identified data routinely collected during clinical management and not requiring specific consent. All patients had provided written informed consent for the transplant procedure and transmission of anonymised data to the EBMT registry. Given the lack of validated diagnostic criteria in this population, we took the pragmatic approach of using a ferritin ≥10000 μg/L as suggestive of actual or threatened cytokine storm/hyperinflammation, in patients with a febrile (and often cytopaenic or coagulopathic) illness with no identified infective pathogens (other than recognised triggers of sHLH) (Table 5).

**Table 5:** Summary of identified cases of sHLH following allogeneic HSCT at Sheffield Teaching Hospitals NHS Foundation Trust from 2014–2018 inclusive

Case ID	Age	Gender (patient/donor)	Diagnosis	Donor information (HLA match, cell source, SIB/VUD)	Transplant conditioning	Date of allogeneic HSCT	DLI details (dates given and dose in CD3^+^cells/kg)	GvHD diagnosis date	GVHD site and grade	GVHD treatment	HLH diagnosis date	*H* Score at diagnosis (probability of HLH)	HLH-2004 score at diagnosis	Putative HLH trigger	Highest serum ferritin (μg/L)	Max EBV PCR (copies/mL) and treatment	CMV and other herpes viruses	HLH treatment	outcome
1	54	M/F	CML	10/10 HLA match, PBSC, SIB	RIC (Flu/Bu/ATG)	09/10/2014	N/A	07/01/2015—skin biopsy. 14/01/2015—sigmoid biopsy	Gut 2, skin 1	IV and PO corticosteroids, CSA, etanercept, ECP	19/12/2015	175 (61%)	3	EBV and chest sepsis	>100,000	24,000. No treatment	Low level CMV (71 copies/mL); settled spontaneously without treatment	(1) IV Dexamethosone and CSA (2) IVIG/IVMP (3) Anakinra 100 mg OD maintained on PO corticosteroid and anakinra 100 mg alternate days	Alive
2	72	M/F	AML (post-ET)	10/10 HLA match, PBSC, VUD	RIC (FMC)	13/04/2016	Dose #1 16/2/17 (0.5 × 10^6^)	24/03/2017—skin (no biopsy). 04/10/2017—sigmoid biopsy	Gut 3, skin 3	IV, PO and topical corticosteroids, CSA, etanercept, ECP	28/10/2017	213, (94%)	4	EBV PTLD	25,468	224,000. 1 dose IV rituximab	Not detected	(1) IVMP/IVIG (2) Anakinra 100 mg OD (3) CSA (4) Further IVMP/IVIG and anakinra 100 mg BD	Died at day +586 post-HSCT
3	43	F/M	MDS	10/10 HLA match, PBSC, VUD	RIC (FMC)	09/03/2017	N/A	09/07/2017—sigmoid biopsy	Gut 2, skin 3	IV, PO and topical corticosteroids, CSA, MMF, etanercept, ECP	01/09/2018	112, (3%)	1	GVHD	28,231	No reactivation	Not detected	(1) IVMP/IVIG (2) Anakinra 100 mg OD (3) CSA (4) Further IVMP/IVIG and anakinra 300 mg OD	Died at day +348 post-HSCT
4	57	F/M	AML	10/10 HLA match, PBSC, SIB	RIC (FMC)	27/02/2015	N/A	08/04/2015 —sigmoid biopsy. 08/04/2015 —skin biopsy	Gut 3, skin 3	IV and PO corticosteroids, CSA, MMF, ECP	16/08/2016	165, (46%)	3	Chest sepsis	33,952	819,000. 1 dose IV rituximab	Not detected	(1) IVMP/IVIG (2) Anakinra 100 mg OD (3) Etoposide	Died at day +561 post-HSCT
5	42	M/M	AML	10/10 HLA match, PBSC, SIB	RIC (FMC)	22/04/2014	Dose #1 15/09/2014 (1 × 10^6^); Dose #2 19/11/2014(5 × 10^6^)	Not diagnosed with GvHD	N/A	N/A	10/12/2017	202, (89%)	4	EBV PTLD	48,639	156 000. 6 doses of IV and IT rituximab	Not detected	(1) IVMP/IVIG (2) Anakinra 100 mg OD (3) Anakinra 200 mg BD and further IVIG	Died at day +1 352 post-HSCT
6	58	M/M	AML	10/10 HLA match, PBSC, VUD	RIC (FLAMSA-BU)	27/07/2018	N/A	06/09/2018 —skin biopsy, 13/09/2018 —sigmoid biopsy	Gut 4, skin 2	IV and PO corticosteroids, CSA, MMF, ECP	31/08/2018	165, (46%)	4	GvHD	11,750	No reactivation	Moderate level CMV reactivation (721 copies/mL)	(1) CSA/MMF (2) IVMP/IVIG (3) Anakinra 200 mg BD	Died day +60 post-HSCT

*M* male, *F* female, *CML* chronic myeloid leukaemia, *AML* acute myeloid leukaemia, *ET* essential thrombocythaemia, *MDS* myelodysplastic syndrome, *EBV* Epstein–Barr virus, *CMV* cytomegalovirus, *PTLD* post-transplant lymphoproliferative disorder, *GvHD* graft versus host disease, *HLA* human leucocyte antigen, *VUD* volunteer unrelated donor, *SIB* sibling donor, *RIC* reduced intensity conditioning, *Flu/Bu/ATG* fludarabine, busulphan, anti-thymocyte globulin, *FMC* fludarabine, melphalan, alemtuzumab, *FLAMSA-BU* fludarabine, cytarabine, amsacrine, busulphan, ATG, *MMF* mycophenylate mofetil, *HSCT* haematopeitic stem cell transplant *DLI* donor leucocyte infusion, *N/A* not applicable, *IV* intravenous, *PO* per oral, *CSA* cyclosporin A, *ECP* extracorporeal phototherapy, *PCR* polymerase chain reaction, *IT* intrathecal, *IVMP* intravenous methylprednisolone, *IVIG* intravenous immunoglobulins, *OD* once daily, *BD* twice daily

Six cases of sHLH presented up to 3 years following first allogeneic HSCT for myeloid malignancies (AML, chronic myeloid leukaemia and myelodysplastic syndrome) between 1st January 2014 and 31st December 2018 (Table 5). This gives an sHLH frequency of 3% i.e. 6 of a total of 202 first allogeneic HSCT procedures undertaken in our unit in patients ≥16 years over the same period, of which 145 were for myeloid disorders. None of these patients had HLH pre-transplant.

The first patient (Case 1) survived after treatment with anakinra. Integrated rheumatology–haematology collaboration in relation to suspected sHLH was maintained thereafter for other suspected cases. This included early screening with ferritin and prompt rheumatological review, with combined management of cases fulfilling criteria for sHLH by accepted classifications. Notwithstanding this proactive approach, five subsequent patients died despite prompt recognition and targeted treatment of sHLH. Of note, five of these six patients had severe GvHD, refractory to standard therapy before developing cytokine storms, and four had associated EBV reactivation/infection. All patients had a fully 10/10 HLA-matched transplant for a myeloid malignancy, but otherwise there were no obvious commonalities e.g. within HLA-types, or other factors.

We did not diagnose sHLH in any of the 385 patients who underwent a first (*n* = 309) or subsequent (*n* = 76) autologous HSCT in our unit over the same period, for which predominant indications were multiple myeloma, lymphoma, autoimmune diseases and solid tumours. We did not diagnose sHLH following first allogeneic HSCT in adults > 16 years for other non-myeloid indications (*n* = 57), which were either lymphoproliferative (*n* = 50) or non-malignant (*n* = 7).

## Discussion

Despite recent major improvements in outcomes in other settings (fHLH and sJIA), sHLH is an under-recognised and life-threatening complication following HSCT with major unmet needs in diagnosis and clinical management. In particular, there are no agreed diagnostic criteria or treatment guidelines for sHLH in the post-HSCT population. We have therefore reviewed the current literature and reported recent experience from our own adult unit in applying early diagnostic and targeted approaches from current rheumatological practice.

We identified six adults with sHLH presenting up to 3 years following allogeneic HSCT using classification and diagnostic criteria used in rheumatological practice. Given the lack of validated diagnostic criteria in this population, we took the pragmatic approach of using a ferritin ≥10000 μg/L as suggestive of actual or threatened cytokine storm/hyperinflammation, in patients with a febrile, and often cytopaenic or coagulopathic, illness with no identified infective pathogens. An alternative, recommended approach to diagnosing adult HLH, is to use the HLH-2004 criteria, in conjunction with clinical judgement. According to the recent consensus, the presence of ≥5 of the HLH-2004 criteria is suggestive of HLH [4]. Of concern, none of our six patients fulfilled >4/8 criteria at the point of diagnosis of HLH so relying on these criteria alone in the post-HSCT population may result in diagnostic and treatment delay, justifying further study into accurate diagnostics. With an apparent incidence of 3% of our total allogeneic HSCT activity over the same time period, along with a high mortality there are significant implications for complexities and costs of clinical management in allogeneic HSCT practice.

We propose that sHLH should be considered carefully in any acutely or chronically unwell allogeneic HSCT recipient, especially those with GVHD, EBV or other viral reactivation, who develop an unexplained, culture-negative febrile illness, pancytopenia or coagulopathy. Despite a proactive and prompt multidisciplinary approach and use of modern sHLH treatment, including IL-1 blockade, which has revolutionised the management of sHLH/MAS in patients with sJIA and SLE and is increasingly reported to be used in both children and adults with sHLH, post-HSCT sHLH contributed heavily to non-relapse mortality in five of six patients (83%). This highlights the need for further study and critical evaluation of this area.

The close relationship of sHLH with GvHD is a common theme and both sHLH and GVHD could be part of a “hyperinflammatory” spectrum. Persistent GvHD is reported in children with sHLH post-HSCT, and we have provided further evaluation in adults [35]. Significantly, we also found co-existing EBV reactivation in four of six patients, which is consistent with reports of infections triggering sHLH post-HSCT [72, 73]. Exposure to GvHD may lead to a “trained immunity” phenomenon, predisposing to hyperinflammation in the form sHLH once a response is triggered, such as by EBV or other infection/reactivations, or other factors [74]. This “alloreactivity” hypothesis is supported by the lack of association of sHLH with autologous HSCT for lymphoproliferative and autoimmune disorders, even though they are recognised triggers for sHLH in settings outside of HSCT. However, whether sHLH is a phenomenon seen more commonly in adults undergoing allogeneic HSCT for myeloid disorders, as suggested by our series, remains to be determined, particularly as other experience varies, especially in paediatric HSCT [33–39].

In conclusion, sHLH is a serious but under-recognised complication following allogeneic HSCT, with high mortality. As with other contexts (such as rheumatological disease), there appears to be a major overlap of sHLH with a background of uncontrolled inflammation and immune dysregulation, such as GvHD, and infective triggers e.g. EBV. On a practical level, serum ferritin is a useful, cheap and readily available biomarker in identifying patients at risk, especially when ≥10000 μg/L, and may have discriminatory value in distinguishing sHLH from GvHD [50]. We therefore propose that routine screening with ferritin post-HSCT identify sHLH earlier, especially in patients with GvHD in whom EBV and other viral reactivations are being detected by routine polymerase chain reaction. Other biomarkers, such as NK cell activity and sIL-2r/CD-25 status are emerging and could be evaluated for their sensitivity and specificity in this setting [45, 54].

Clinically, with an estimated incidence of 3% in our allogeneic HSCT recipient population and a high mortality despite modern treatment approaches, there are major unmet needs in this area. Alongside of these discussions, the advent of chimeric antigen receptor (CAR) T-cell therapy also brings with it the risk of sHLH and other cytokine storm-related complications, requiring close collaboration between haematologists, rheumatologists, critical care specialists and immunologists [75, 76]. Further studies in all ages of patients are warranted. Larger retrospective studies, for example, via the EBMT registry, are a means to more comprehensively characterise the frequency, severity and outcomes following mostly “ad hoc” treatment of sHLH in HSCT and cellular therapy recipients. However, prospective studies, including validation of diagnostic markers and clinical trials of cytokine blockade, are urgently required to establish effective identification and management of sHLH following HSCT, CAR T-cell and other cellular therapies.

## Supplementary information

Table 5 (word document at reviewer's request)
