# Supplementary material for: Haemophagocytic lymphohistiocytosis (HLH) following allogeneic haematopoietic stem cell transplantation (HSCT)—time to reappraise with modern diagnostic and treatment strategies?
Source: Bone Marrow Transplant. 2019 Aug 27;55(2):307–16. doi: 10.1038/s41409-019-0637-7 (PMC6995779; doi:10.1038/s41409-019-0637-7)
Supplement: Supplementary file 1 — Table 5 (word document at reviewer's request) [file 41409_2019_637_MOESM1_ESM.docx]

| **Case ID** | **Age** | **Gender (patient**  **/donor)** | **Diagnosis** | **Donor Information (HLA match, cell source, SIB/VUD)** | **Transplant conditioning** | **Date of allogeneic HSCT** | **DLI details (dates given and dose in CD3^+^cells/kg)** | **GvHD Diagnosis Date** | **GVHDSites and grade** | **GVHD Treatment** | **HLH Diagnosis Date** | **H Score at diagnosis (probability of HLH)** | **HLH-2004 score at diagnosis** | **Putative HLH trigger** | **Highest serum ferritin (μg/L)** | **Max EBV PCR (copies/mL) and treatment** | **CMV and other herpes viruses** | **HLH Treatment** | **Outcome** |
| --- | --- | --- | --- | --- | --- | --- | --- | --- | --- | --- | --- | --- | --- | --- | --- | --- | --- | --- | --- |
| 1 | 54 | M/F | CML | 10/10 HLA match, PBSC, SIB | RIC (Flu/Bu/ATG) | 09/10/2014 | N/A | 07/01/2015 - skin biopsy. 14/01/2015 - sigmoid biopsy. | Gut 2, Skin 1 | IV and PO corticosteroids , CSA, etanercept, ECP. | 19/12/2015 | 175 (61%) | 3 | EBV and chest sepsis | >100 000 | 24 000. No treatment. | Low level CMV (71 copies/mL); settled spontaneously without treatment. | 1 – IV Dexamethosone and CSA 2 – IVIG/IVMP 3 – Anakinra 100mg OD Maintained on PO corticosteroids and anakinra 100mg alternate days | Alive |
| 2 | 72 | M/F | AML (post-ET) | 10/10 HLA match, PBSC, VUD | RIC (FMC) | 13/04/2016 | Dose #1 16/2/17 (0.5x10^6^) | 24/03/2017 - skin (no biopsy). 04/10/2017 - sigmoid biopsy | Gut 3, Skin 3 | IV, PO and topical corticosteroids, CSA, etanercept, ECP. | 28/10/2017 | 213, (94%) | 4 | EBV PTLD | 25 468 | 224 000. 1 dose IV rituximab | Not detected. | 1 - IVMP/IVIG 2 – Anakinra 100mg OD 3 – CSA 4 – Further IVMP/IVIG and anakinra 100mg BD | Died at day + 586 post-HSCT |
| 3 | 43 | F/M | MDS | 10/10 HLA match, PBSC, VUD | RIC (FMC) | 09/03/2017 | N/A | 09/07/2017 - Sigmoid biopsy | Gut 2, Skin 3 | IV, PO and topical corticosteroids, CSA, MMF, etanercept, ECP. | 01/09/2018 | 112, (3%) | 1 | GVHD | 28 231 | No reactivation. | Not detected. | 1 – IVMP/IVIG 2 – Anakinra 100mg OD 3 – CSA 4 – Further IVMP/IVIG and anakinra 300mg OD | Died at day +348 post-HSCT |
| 4 | 57 | F/M | AML | 10/10 HLA match, PBSC, SIB | RIC (FMC) | 27/02/2015 | N/A | 08/04/2015 - sigmoid biopsy. 08/04/2015 - skin biopsy. | Gut 3, Skin 3 | IV and PO corticosteroids, CSA, MMF, ECP. | 16/08/2016 | 165, (46%) | 3 | Chest sepsis | 33 952 | 819 000. 1 dose IV rituximab. | Not detected. | 1 – IVMP/IVIG 2 – Anakinra 100mg OD 3 – Etoposide | Died at day +561 post-HSCT |
| 5 | 42 | M/M | AML | 10/10 HLA match, PBSC, SIB | RIC (FMC) | 22/04/2014 | Dose #1 15/09/2014 (1x10^6^);  Dose #2 19/11/2014 (5x10^6^) | Not diagnosed with GvHD | N/A | N/A | 10/12/2017 | 202, (89%) | 4 | EBV PTLD | 48 639 | 156 000. 6 doses of IV and IT rituximab. | Not detected. | 1 – IVMP/IVIG 2 – Anakinra 100mg OD 3 –Anakinra 200mg BD and further IVIG | Died at day +1 352 post-HSCT |
| 6 | 58 | M/M | AML | 10/10 HLA match, PBSC, VUD | RIC (FLAMSA-BU) | 27/07/2018 | N/A | 06/09/2018 - Skin biopsy, 13/09/2018 - Sigmoid biopsy | Gut 4, Skin 2 | IV and PO corticosteroids, CSA, MMF, ECP. | 31/08/2018 | 165, (46%) | 4 | GvHD | 11 750 | No reactivation. | Moderate level CMV reactivation (721 copies/mL). | 1 – CSA/MMF 2 – IVMP/IVIG 3 - Anakinra 200mg BD | Died day +60 post-HSCT |

Key:

M (male), F (female), CML (chronic myeloid leukaemia), AML (acute myeloid leukaemia), ET (essential thrombocythaemia), MDS (myelodysplastic syndrome), EBV (Epstein-Barr virus), CMV (cytomegalovirus) PTLD (post-transplant lymphoproliferative disorder), GvHD (graft versus host disease), HLA (human leukocyte antigen), VUD (volunteer unrelated donor), SIB (sibling donor), RIC (reduced intensity conditioning), Flu/Bu/ATG (fludarabine, busulphan, anti-thymocyte globulin), FMC (fludarabine, melphalan, alemtuzumab), FLAMSA-BU (fludarabine, cytarabine, amsacrine, busulphan, ATG), MMF (mycophenylate mofetil), HSCT (haematopeitic stem cell transplant) DLI (donor leukocyte infusion), N/A (not applicable), IV (intravenous), PO (per oral), CSA (cyclosporin A), ECP (extracorporeal phototherapy), PCR (polymerase chain reaction), IT (intrathecal), IVMP (intravenous methylprednisolone) IVIG (intravenous immunoglobulins), OD (once daily), BD (twice daily)
